# Early warm compress treatment can promote recanalization of vascular embolisms and reduce tissue necrosis after polymethyl methacrylate injection

**DOI:** 10.1038/s41598-023-29043-8

**Published:** 2023-02-01

**Authors:** Yimou Sun, Mengying Jin, Guanhuier Wang, Hongbin Xie

**Affiliations:** Department of Plastic Surgery, Peking University Third Hospital, 49 North Garden Rd., Haidian District, Beijing, 100191 China

**Keywords:** Thrombosis, Experimental models of disease

## Abstract

Injection of fillers for soft tissue augmentation can lead to a variety of complications, among which vascular occlusion caused by intravascular injection of filler will induce severe or permanent damage. The treatment strategies for intravascular embolization caused by injection include warm compress application, but the exact beneficial effects of this therapy have not been confirmed. The purpose of this study is to construct an intravascular injection embolism model and observe the effectiveness of warm and cold compress through a randomized, controlled trial. Thirty rabbit’s sixty ears were randomly divided into warm compress group, cold compress group, and control group. Polymethyl methacrylate (PMMA) was slowly injected into the central ear artery (CEA) to cause vascular embolism. Warm compress and cold compress treatment were performed respectively. The vascular recanalization and other related indexes were observed at 30 min, 1 day, and 7 days after injection, and the tissue necrosis was analyzed at 7 days. In the early stage of vascular embolization, warm compress can immediately promote vascular dilatation, blood circulation and partial blood flow recovery. One day after intravascular injection, warm compress can reduce intravascular embolization and reduce the incidence of tissue necrosis. At 7 days after intravascular injection, the vessels in the cold compress and control groups were still embolized while the percentage of recanalization in the warm compress group was 47.4% (P < 0.000). Early-stage warm compress after intravascular PMMA injection is conducive to recanalization of vascular embolization and reducing tissue necrosis.

## Introduction

The injection of fillers for soft tissue augmentation is a rapidly developing treatment^1^. Research has shown that from 2000 to 2017, soft tissue filling surgery increased by 300% in the United States^2^. Although the incidence of complications is low and most adverse events are mild, the increase in the number of procedures has led to an increase in the number of complications^2–5^.

Mild or transient complications related to filler injection include swelling, edema, erythema, skin paraesthesia, and local infection. Severe or permanent complications include skin necrosis, vision loss, and blepharoptosis^6^. Complications classified as severe or permanent are mainly caused by vascular compromise, which may be induced by partial or complete vascular occlusion caused by the intravascular injection of filler^7^. To avoid serious adverse consequences, practitioners should quickly identify and actively deal with adverse vascular events, especially intravascular embolization^8^. According to some guidelines and expert consensus recommendations, when vascular obstruction is suspected, the injection should be stopped immediately and treatment should be started quickly^5,8,9^. Treatment strategies include hyaluronidase, warm compress application, massage or tapping of the area, and the application of 2% nitroglycerin paste to promote vasodilation^10–12^.

Although a warm compress is recommended as the first-line treatment^10,13,14^, the exact beneficial effects of this therapy for the treatment of filler-induced vascular embolism have not been confirmed. During this study, we conducted a randomized, controlled trial and injected polymethyl methacrylate (PMMA) into rabbit ear arteries to construct a vascular embolism model. Therapy comprising a warm compress and cold compress was applied to observe the effectiveness of the warm compress immediately after injection and during the long term after embolization.

## Methods

### Animals and injected products

Thirty healthy male White rabbits (2.7–3.2 kg) were provided by the experimental animal center at Peking University Health Science Center. In the experimental animal center, rabbits were raised in metal cages under natural light conditions of 22 ± 2 °C and 60 ± 10% relative humidity; they were allowed to eat and drink freely. The PMMA filler (Artecoll; Hafod BV, Rotterdam, the Netherlands) was injected intra-arterially. All procedures performed during this study were approved by the Experimental Animal Ethics Committee of Peking University Health Science Center (no. A2021321) and followed the relevant guidelines for animal care and use. All methods are reported in accordance with ARRIVE guidelines.

### Intravascular injection and treatment

**Figure 1:**
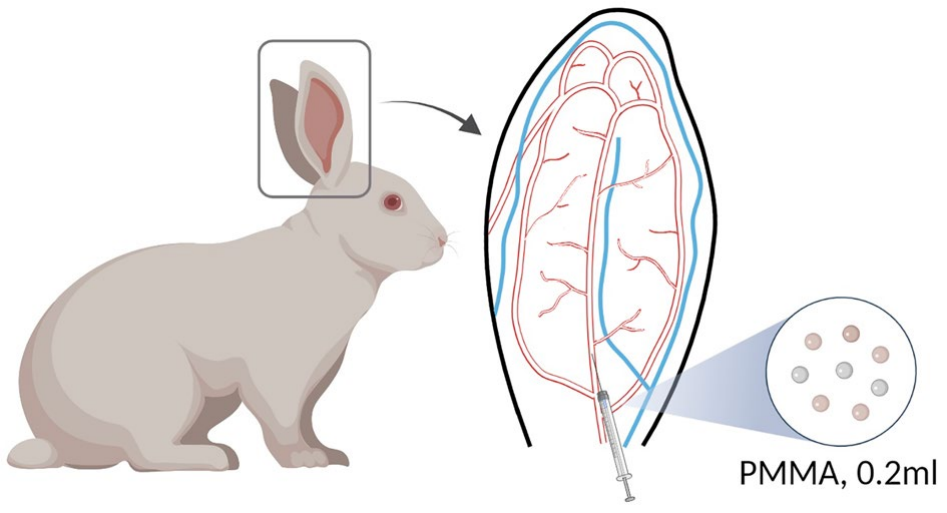
Schematic diagram of intravascular injection in the rabbit ear. Injection site: approximately 1 cm above the bifurcation of the auricular artery. Figure was created on BioRender (https://biorender.com/) and in Procreate application (version 5.2.6; https://procreate.com/).

Three groups were created during this experiment: warm compress group, cold compress group, and control group. Sixty ears of 30 rabbits were randomly assigned to each group using a random number table (n = 20). Zoletil (5 mg/mL) and xylazine (5 mg/mL) was injected subcutaneously at 0.8 mL/kg of body weight to anesthetize the animals. After anesthesia and routine disinfection, the ventral side of the rabbit ear was irradiated with strong light, and the filler was slowly injected in the trunk of the central ear artery (CEA) with a 27-G needle (0.5 mm) from 1 cm above the bifurcation of the auricular artery at a uniform speed (Fig. 1). The injection speed was controlled at approximately 0.2 mL/30 s. One doctor completed the injection for all rabbits, and practiced repeatedly before the injection to ensure that the speed was uniform. During injection, the formation of an embolus and characteristics of the changes in blood flow were carefully observed and recorded. Warm and cold compress were performed immediately after injection. Before injection, all rabbit ears were at room temperature, and the warm compression device and cold compression device were adjusted to the set temperature in advance to ensure that the rabbit ears were treated immediately after injection. During the application of the warm compress, the rabbit ears were covered with two small heating blankets that were secured with adhesive tape. The temperature of the heating blanket was set to 40 °C. The warm compress was applied for 30 min. The surface temperatures of the rabbit ears at 10 min, 20 min, and 30 min after injection were measured and recorded. The average temperature was calculated using the three aforementioned measurements and was considered the final temperature of the warm compress. During the application of the cold compress, the rabbit ears were covered with a cold bag containing a cold water mixture that was secured with adhesive tape. The cold compress was applied for 30 min. The surface temperatures of the rabbit ears at 10 min, 20 min, and 30 min after injection were measured and recorded. The average temperature was calculated using the three aforementioned measurements and was considered the final temperature of the cold compress. The rabbit ears in the warm compress group were maintained at 41.88 ± 1.18 °C; those in the cold compress group were maintained at 9.94 ± 1.67 °C during observation. Rabbits in the control group were kept in a room with a normal temperature (20–22 °C) for 30 min.

### Observation of CEA embolism and skin necrosis

**Figure 2:**
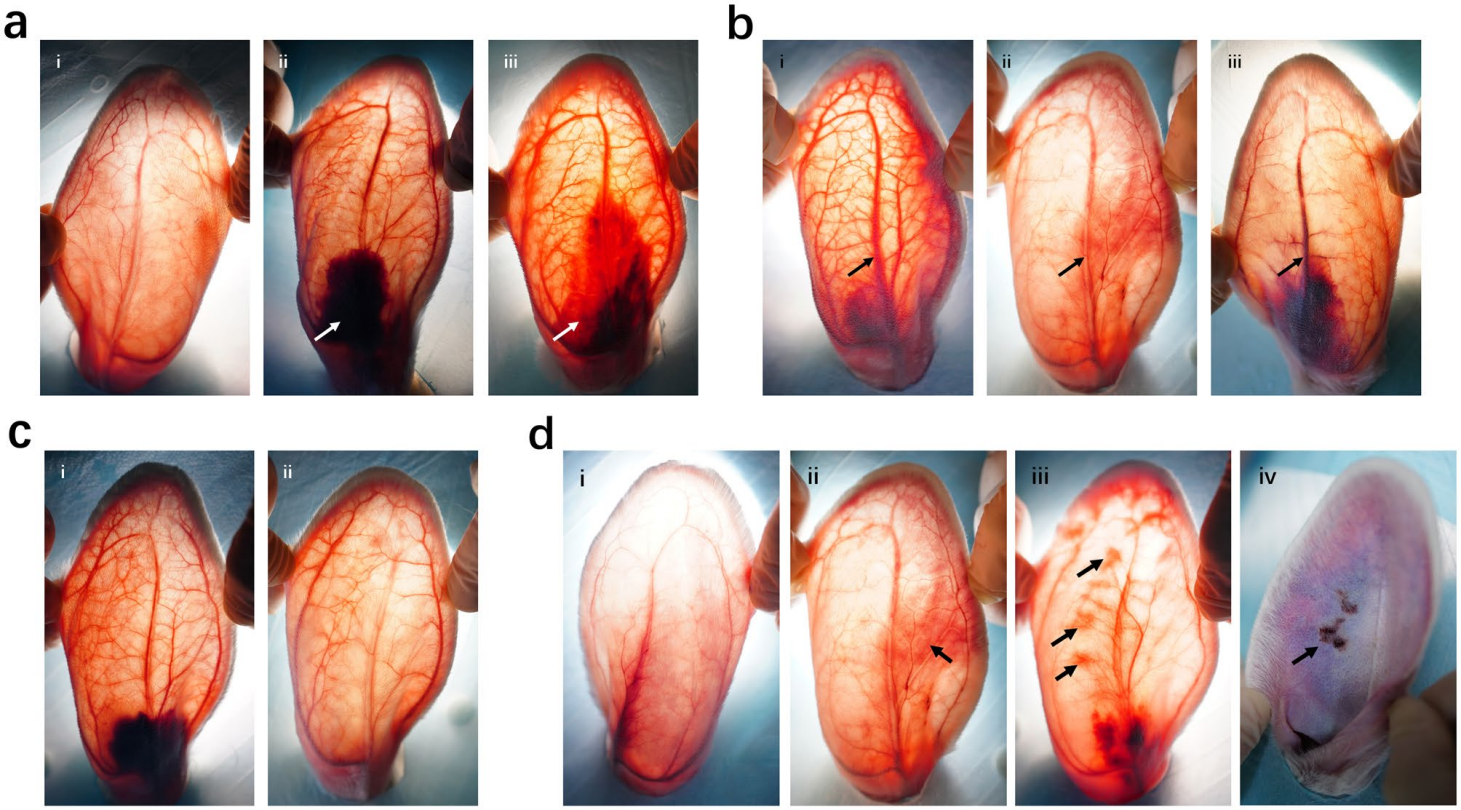
The diagram of observation indexes. (**a**) The formation and regression of hematoma. i: no hematoma; ii: arrow: hematoma; iii: arrow: part of the hematoma regression. (**b**) Vascular recanalization and different types of thrombosis. i: vascular recanalization; ii: arrow: no recanalization with white thrombus; iii: arrow: no recanalization with red thrombus. (**c**) Skin color. i: ruddy; ii: pale. (**d**) Different degrees of tissue necrosis. i: normal; ii: mild necrosis; arrow: erythema. iii: moderate necrosis; arrows: erythema. iv: severe necrosis; arrow: ulceration. Figure was created in Adobe Illustrator software (version 22.1; https://www.adobe.com/products/illustrator.html).

The vascular embolism and skin necrosis of rabbit ears were observed and recorded using a camera (Sony A7RM4 with a 24–70 f2. 8 lens; Sony, Tokyo, Japan) at 30 min, 1 day, and 7 days after injection. The observation indexes included skin color, formation and regression of hematomas, vascular recanalization, cord-like induration, thrombus type, and tissue necrosis (Fig. 2).

The skin color of each rabbit ear was observed and recorded at 30 min after injection. The skin color at each timepoint was classified as ruddy or pale. The formation and regression of hematomas in the three groups at 30 min and 1 day after injection were calculated and analyzed.

Vascular recanalization could be intuitively observed because recanalized CEAs were red and full of flowing blood, and non-recanalized CEAs were filled with PMMA gel and thrombus. Recanalization of each ear was distinguished as “yes” or “no” and recorded. The percentage of recanalization of each group was calculated at 30 min, 1 day, and 7 days after injection.

Cord-like induration was judged by touching those vessels with high tension caused by temporary congestion of PMMA gel and thrombus. The incidences of cord-like induration in the three groups at 1 day and 7 days after injection were calculated and analyzed.

Two thrombus types were classified to obtain a better description of the thrombus characteristics. A white thrombus was caused by congested PMMA gel. A red thrombus was caused by coagulated blood. The thrombus type of each ear could be intuitively observed and recorded. The incidences of white thrombi and red thrombi in the three groups at 1 day and 7 days after injection were calculated and analyzed.

Abnormal conditions, such as reticularis, erythema, and ulceration, were recorded. Skin necrosis was observed and classified as four degrees: normal, no obvious abnormality of the skin tissue; mild necrosis, erythema with fewer than three spots; moderate necrosis, erythema with three or more spots; and severe necrosis, eschar formation and ulceration of any degree. The proportions of each degree in the three groups at 1 day and 7 days after injection were calculated and analyzed. To avoid bias, each index was discussed and classified by three experienced surgeons.

### Histological examination

The rabbits were killed 7 days after injection. Ear tissues were fixed with 4% paraformaldehyde at room temperature (26 °C) overnight. Samples were passed through serial concentrations of ethanol for paraffin embedment. Hematoxylin and eosin (H&E) staining were performed after sectioning. Following dewaxing and rehydrating, the sections were stained with hematoxylin dye solution for 20 min at room temperature, followed by eosin staining for 1 min at room temperature^15^.

### Statistical analysis

Data were analyzed using IBM SPSS statistics 24 for Windows. The chi-square test or Fisher test was used to analyze descriptive statistics and Bonferroni correction was applied. The measured rabbit ear temperature of the warm compress group and the cold compress group were expressed as mean ± standard deviation. Bonferroni-corrected P < 0.05 was considered statistically significant.

### Ethical approval

The project was performed after the ethical examination of experimental animal ethics committee in our hospital (approval No. A2021321).

### Human and animal rights

All applicable institutional and/or national guidelines for the care and use of animals were followed.

### Informed consent

For this type of study informed consent is not required.

## Results

### Construction of the intravascular embolization model

**Figure 3:**
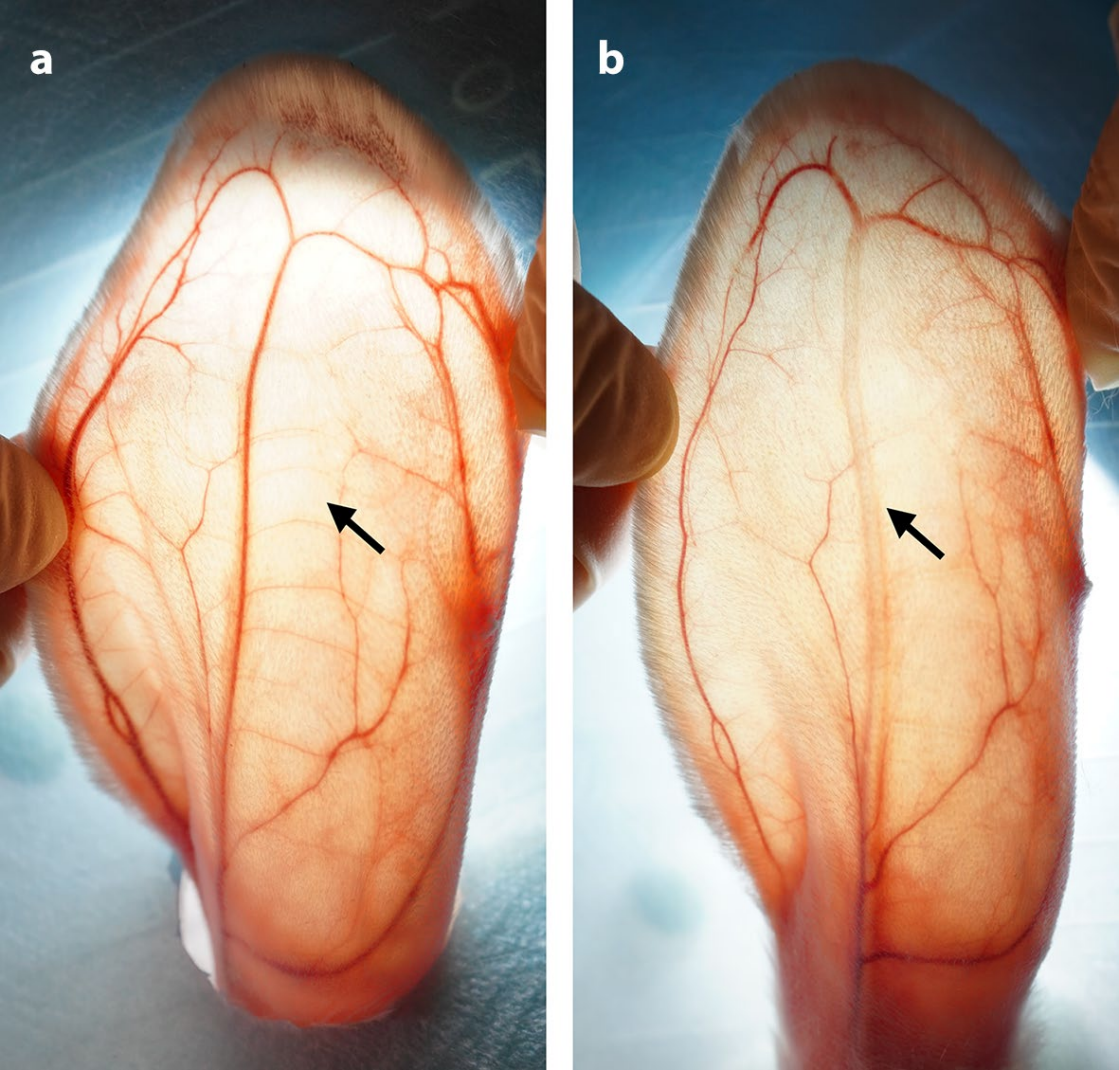
Immediate changes after intravascular filler injection. (**a**) before injection; (**b**) after injection. Arrow: the trunk of the central ear artery. Figure was created in Adobe Illustrator software (version 22.1; https://www.adobe.com/products/illustrator.html).

When the PMMA filler was injected into the CEA, it was clearly observed that the blood flow in the CEA was pushed from the proximal end to the distal end, and that the blood vessel immediately became translucent. Simultaneously, the branching vessels on both sides of the CEA were difficult to visualize and became pale (Fig. 3).

### Immediate effects of the warm compress on filler-induced vascular embolism

**Figure 4:**
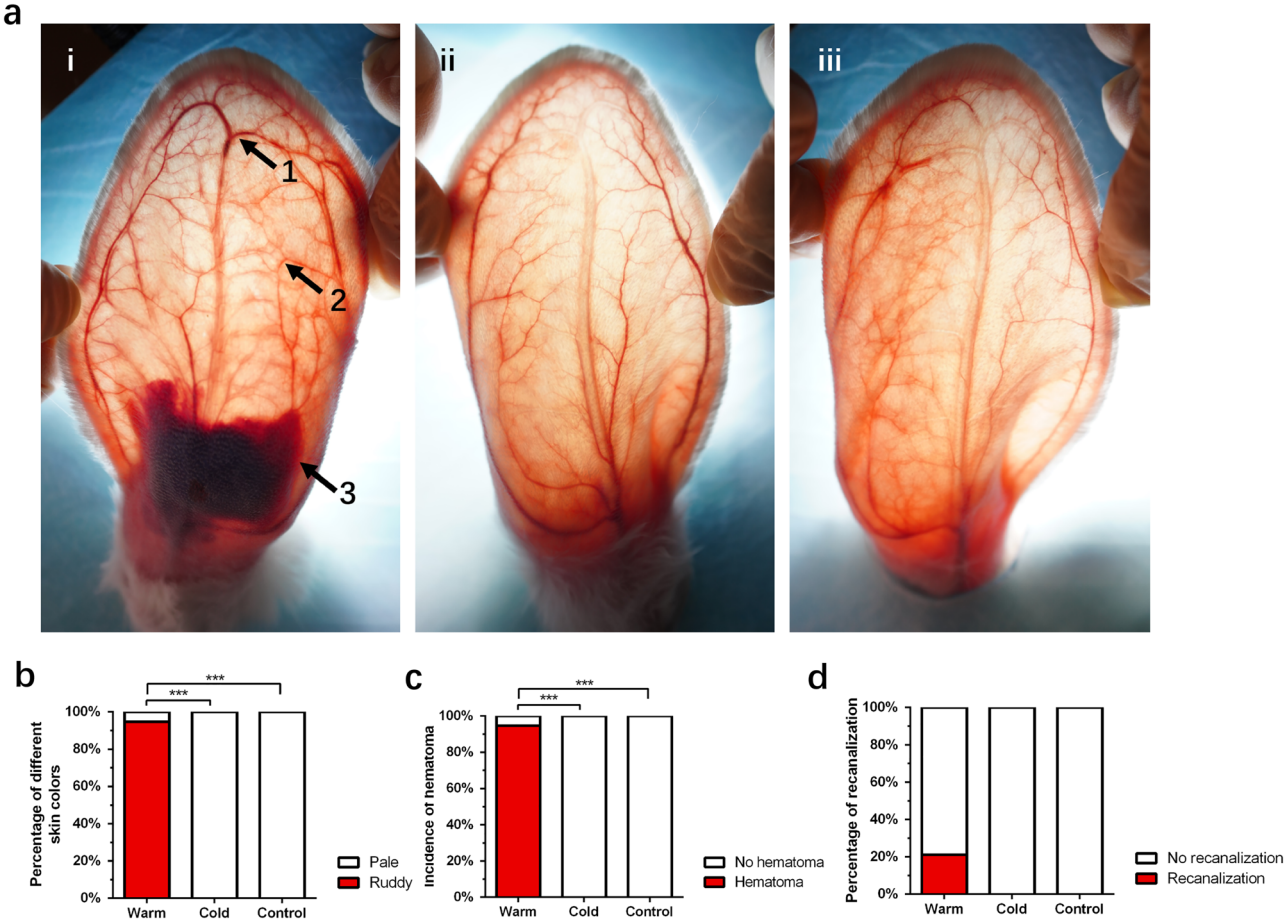
Changes in three groups at 30 min after intravascular injection. (**a**) i: warm compress. Arrow 1: vascular recanalization and filling; Arrow 2: expansion of the peripheral branch blood vessels; Arrow 3: hematoma. ii: cold compress. iii: control group. (**b**) The skin color changes in different groups at 30 min after intravascular injection. (**c**) Incidence of hematoma in different groups at 30 min after intravascular injection. (**d**) Percentage of vascular recanalization in different groups at 30 min after intravascular injection. ***P < 0.000. Figure was created in Adobe Illustrator software (version 22.1; https://www.adobe.com/products/illustrator.html) and GraphPad Prism software (version 9.4.1; https://www.graphpad.com/scientific-software/prism/).

At 30 min after injection, compared with the cold compress and control groups, the warm compress group showed obvious expansion of the peripheral branch blood vessels. Simultaneously, we observed that although there was still an embolism in the main trunk of the CEA, some vascular recanalization and filling had appeared in the distal end (Fig. 4a).

It was observed that most (94.7%) of the ear skin color in the warm compress group became ruddy; however, this was not observed in the cold compress and control groups (Fig. 4b).

Additionally, we observed a significant increase in the incidence of hematoma in the warm compress group (94.7%); however, there was no hematoma formation in the cold compress and control groups (Fig. 4c). We also noticed that 21.1% of the blood vessels in the warm compress group were recanalized compared with 0% in the cold compress and control groups; however, there was no significant difference (Fig. 4d).

### Short-term effects of the warm compress on filler-induced vascular embolism

**Figure 5:**
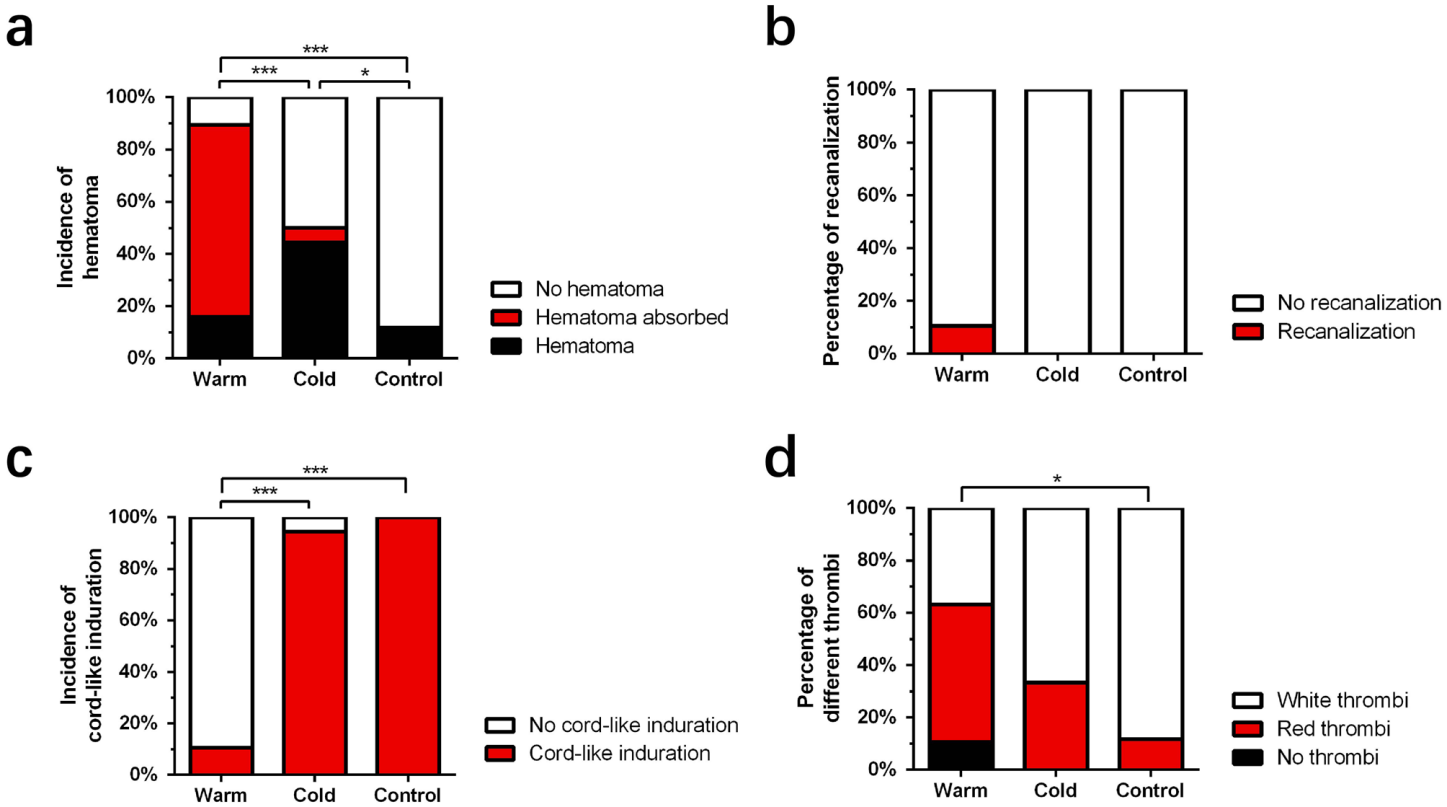
Changes in three groups at 1 day after intravascular injection. (**a**) Incidence of hematoma in different groups 1 day after intravascular injection. (**b**) Percentage of vascular recanalization in different groups 1 day after intravascular injection. (**c**) Incidence of cord-like induration in different groups 1 day after intravascular injection. (**d**) Percentage of different embolisms in different groups 1 day after intravascular injection. *P < 0.05; ***P < 0.000. Figure was created in Adobe Illustrator software (version 22.1; https://www.adobe.com/products/illustrator.html) and GraphPad Prism software (version 9.4.1; https://www.graphpad.com/scientific-software/prism/).

One day after vascular embolization, we noticed that the overall color of rabbit ears returned to ruddy, with no obvious difference among the three groups. The hematomas in the warm compress group were significantly absorbed compared with that during the previous day, accounting for an incidence of 73.7%. Hematomas began to appear in the cold compress group and control group, with incidences of 44.4% and 11.8%, respectively (Fig. 5a). The hematoma incidences 1 day after injection in the warm and cold compress groups were higher than that in the control group (P < 0.05), which suggested that although the immediate effect of the cold compress was the constriction of blood vessels, it still seemed to promote the delayed occurrence of hematoma.

At this stage, the incidence of recanalization in each group was still low, and there was no significant difference (Fig. 5b). However, only 10.5% of the warm compress group had intravascular cord-like induration; in the cold compress group and control group, the rates of intravascular cord-like induration were 94.4% and 100%, respectively (Fig. 5c).

To further clarify thromboembolisms in the different groups, we observed and counted the thrombosis types in each group. The statistical results showed that without intervention, intravascular injection was more likely to produce a white thrombus (88.2%). Compared with the control group, the incidence of red thrombi in the warm compress group was higher (52.6%) and the incidence of white thrombi was lower (36.8%) (p < 0.05); however, there was no significant difference between the cold compress group and the warm compress and control groups (Fig. 5d). In addition, 61.1% of the cold compress group had different degrees of tissue necrosis; however, 21.1% of the warm compress group and 29.4% of the control group had different degrees of tissue necrosis. The results showed that temperature had an effect on tissue necrosis, and the incidence of necrosis in the warm compress group was the lowest (P < 0.05). There was no significant difference in the degrees of necrosis among the three groups (Table 1).

**Table 1:** Tissue necrosis 1 day after intravascular injection.

Group	Normal	Mild	Moderate	Severe	Total necrosis
Warm	15 (78.9%)	3 (15.8%)	1 (5.3%)	0 (0%)	21.1%*
Cold	7 (38.9%)	7 (38.9%)	3 (16.7%)	1 (5.6%)	61.1%
Control	12 (70.3%)	3 (17.6%)	1 (5.9%)	1 (5.9%)	29.4%

*P < 0.05 (warm group vs. cold group).

### Effects of the warm compress at 7 days after injection and related histopathological changes

**Figure 6:**
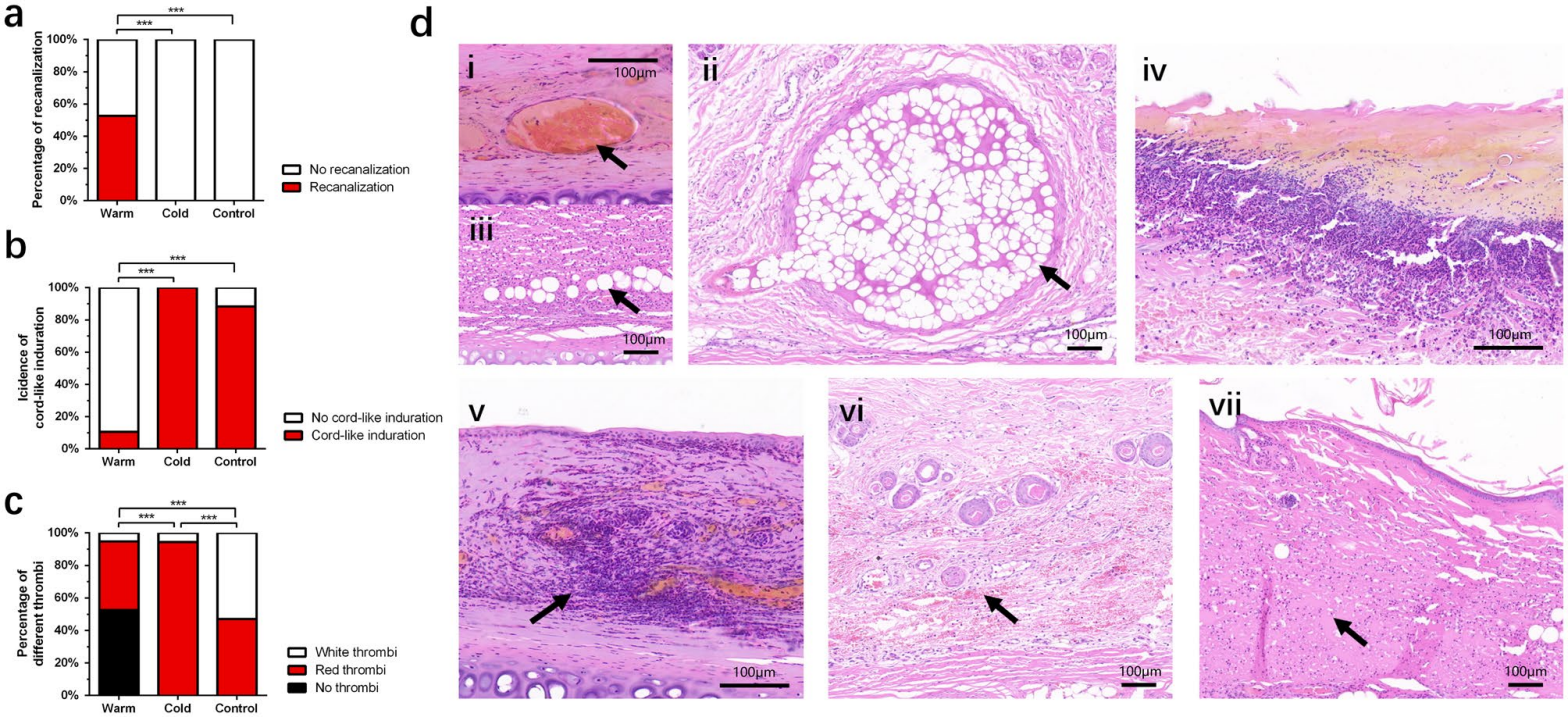
Observation indexes and histopathological changes in 3 groups at 7 days after intravascular injection. (**a**) Percentage of vascular recanalization in different groups 7 days after intravascular injection. (**b**) Incidence of cord-like induration in different groups 7 days after intravascular injection. (**c**) Percentage of different embolisms in different groups 7 days after intravascular injection. (**d**) Histopathological changes 7 days after intravascular injection (H&E stanning). i: arrow: red thrombus composed of red blood cells and light red amorphous substances; ii: arrow: white thrombus composed of a large number of PMMA microspheres; iii: arrow: PMMA microspheres; iv: ulceration; v: erythema, arrow: inflammatory cell infiltration. vi: arrow: small amount of subcutaneous red blood cell infiltration; vii: arrow: severe hematoma. Scale bar: 100 μm. Figure was created in Adobe Illustrator software (version 22.1; https://www.adobe.com/products/illustrator.html) and GraphPad Prism software (version 9.4.1; https://www.graphpad.com/scientific-software/prism/).

At 7 days after injection, the overall skin color of all rabbit ears was ruddy, except for the necrotic site. The vessels in the cold compress group and control group were still embolized; however, 47.4% of the warm compress group had vascular recanalization (Fig. 6a, P < 0.000), indicating that warm compress can promote vascular recanalization. The effects of the warm compress require time to occur. Correspondingly, we observed that the proportion of intravascular cord-like induration in the warm compress group was 10.5%, which was still far lower than that in the cold compress group (100%) and control group (88.2%) (Fig. 6b).

At this stage, the proportions of red thrombi and white thrombi in the control group were similar (47.1% and 52.9% respectively). However, in the warm and cold compress groups, the proportions of red thrombi were much higher than those of white thrombi (Fig. 6c). The histopathological results showed that the red thrombus mainly comprised red blood cells and light red amorphous substances (Fig. 6d-i), and that the white thrombus mainly comprised a large amount of PMMA gel seen as transparent and refractive microspheres with a diameter of approximately 40 μm (Fig. 6d-ii). The PMMA microspheres could be observed in vascular branches deep in the epidermis as well (Fig. 6d-iii). At 7 days after injection, there was no significant difference in tissue necrosis among the three groups (Table 2). There was no severe necrosis in the warm compress group.

**Table 2:** Tissue necrosis 7 days after intravascular injection.

Group	Normal	Mild	Moderate	Severe	Total necrosis
Warm	4 (21.1%)	3 (15.8%)	12 (63.2%)	0 (0%)	78.9%
Cold	5 (27.8%)	3 (16.7%)	6 (33.3%)	4 (22.2%)	72.2%
Control	6 (35.3%)	3 (17.6%)	6 (35.3%)	2 (11.8%)	64.7%

The histopathological changes showed that skin ulcerations occurred in the cold compress group and control group (Fig. 6d-iv), but that only erythema was observed in the warm compress group (Fig. 6d-v). In the warm compress group, a small amount of subcutaneous red blood cell infiltration was observed in some rabbit ears and no large-scale hematoma formation occurred (Fig. 6d-vi). Hematoma infiltration in the cold compress group was more severe (Fig. 6d-vii).

## Discussion

Vascular compromise is one of the most serious complications of filler injections and can lead to a series of problems, such as visual field loss, blindness, and skin necrosis^9^. It is believed that filler injection-induced vascular compromise may be caused by the following mechanisms^16^: direct vascular occlusion with fillers; fillers compressing blood vessels or vasospasm resulting in ischemic changes in tissues; and, for some late-onset vascular complications, although the initially injected fillers may not be sufficient to completely block the blood vessels, blood vessels are subsequently blocked by platelet aggregation. Regardless of the mechanism, when vascular compromise occurs, the tissue will become pale because of ischemia, and reticularis caused by hypoxia will occur. Further infections and different degrees of tissue necrosis may occur^17^. To avoid some irreversible consequences, it is important to identify vascular embolism early and deal with it quickly. Clinicians should first perform treatment to restore perfusion; then, they must support healing as needed. Local heating of the skin produces vasodilation and promotes blood flow^18^; therefore, many believe that the early application of a warm compress has certain therapeutic effects^8,19–21^. However, no animal experiments have confirmed that a warm compress is conducive to the recovery of vascular embolism. Although not recommended, a cold compress can reduce the local tissue metabolic rate and increase the tolerance to ischemia, which can help treat injection embolization^22^. Therefore, we performed this study to explore the therapeutic effects of the application of a warm compress and a cold compress on filler-induced vascular embolism.

The response of the blood flow to local heating results in continuously dilated blood vessels and increased blood flow until a plateau is reached^23^. These responses were significant with rapid local heating, but not with slow local heating^24^. Our results showed that a warm compress can promote vasodilation, blood circulation, and partial blood flow recovery during the early stage of filler-induced vascular embolism. In the experiment, we observed that the color of untreated rabbit ears was ruddy without embolization. At 30 min after intravascular injection, the skin color of rabbit ears in the cold compress group and the control group became pale, whereas that in the warm compress group turned ruddy (94.7%). This result suggests that in the control group, the pale color of skin is the immediate effect of vascular embolism. Skin exposed in low temperature may appear pale^25^. The color change in the cold compress group may be the dual effect of vascular embolism and lower temperature promoting vasoconstriction. Our conclusion is that in the case of embolism, compared with the control group, warm compress can promote the skin color of rabbit ears to restore ruddy while the cold compress did not have a similar effect. In the warm compress group, the collateral circulation provided some support for the ischemic area, and the blood flow of some small vessels recovered. Interestingly, the incidence of ruddy skin and the incidence of hematoma are identical (94.7%) in the warm compress group. But there was one ruddy rabbit ear without hematoma, while there was one pale rabbit ear with hematoma, which indicated that there was a correlation between the two indexes, but not completely. Although a warm compress will lead to a higher incidence of hematoma, these hematomas can be absorbed during the early stage and will not have long-term adverse effects.

The incidence of cord-like induration in the warm compress group was significantly lower than that in the cold compress group and control group at 1 day and 7 days after intravascular injection, indicating that the warm compress reduced intravascular filler congestion. The recanalization of blood vessels is still relatively difficult during the early stage. There was no significant difference between the control group and cold compress group at 30 min after intravascular injection, and recanalization did not occurred in all of the cases. However, recanalization occurred in four cases in the warm compress group and three cases of them involved repeat embolization after 1 day. For these 3 cases, 2 of them had no recanalization at the later time point, recanalization occurred in the remaining 1 case at 7 days after injection. At 1 day after injection, there were 2 cases of vascular recanalization in the warm compress group, of which 1 case was still recanalized at the later time point, and the other case involved repeat embolization at 7 days after injection. These data may indicate that vascular recanalization in the acute stage and early stage (1 day) after warm compress application is not necessarily reliable, possibly because the internal filler cannot completely fill the vascular lumen after vascular dilation, resulting in partial blood filling and lasting for a period of time. However, when the warm compress application is stopped, the blood vessels will gradually contract and platelet aggregation will occur, leading to blood vessel embolization^16^. Nevertheless, we observed a percentage of recanalization of 47.4% at 7 days after injection in the warm compress group, which was much higher than the rates in the cold compress group (0%) and control group (0%). This shows that during the long term, warm compress application has an obvious beneficial effect of promoting vascular recanalization. It is noteworthy that of the 10 rabbit ears recanalized at 7 days, 8 cases of them occluded at the previous two time points. This indicates that there is a possibility of subsequent recanalization after active intervention of vascular embolism, and it also suggests that we can consider a longer period of warm compress treatment. In conclusion, it is necessary to carry out early and continuous intervention on embolic vessels in clinical practice.

In addition to the direct observation indexes, such as intravascular cord-like induration and vascular recanalization, necrosis, as a severe delayed complication, is related to skin ischemia caused by filler embolism^26^. Based on previous studies, we divided the degree of skin necrosis into normal, mild, moderate, and severe categories^10^. The results showed that the incidence of tissue necrosis in the warm compress group was lower during the early stage, but that there was no significant difference when compared with the control and cold compress groups at 7 days after intravascular injection. At 7 days after embolization, although the percentage of recanalization of the main blood vessels in the cold compress group and control group were 0%, most rabbit ears did not have severe necrosis. This may have occurred because of the abundant collateral circulation of rabbit ears and strong tolerance to ischemia. If an organ or tissue is supplied by a main blood vessel and lacks sufficient collateral circulation, then there will be serious consequences when the major blood vessel is completely embolized. For example, embolism of the human central retinal artery will lead to blindness. The percentage of recanalization of blood vessels is particularly important and reflects the importance of the warm compress. Although there were no significant differences in statistics, there was no severe necrosis, such as skin ulcers, in the warm compress group. Therefore, a warm compress can improve tissue necrosis caused by early vascular embolism and reduce the occurrence of long-term severe skin necrosis to a certain extent.

Although early warm massage may promote vasodilation, local heating will also increase the metabolic rate of tissue, thus reducing the ischemic tolerance of tissue^22^. Therefore, some clinical studies have adopted the method of warm massage during the first 30 min after hyaluronidase injection (the specific temperature is unknown), followed by local cooling with a gauze pad soaked with antibiotic saline solution^22^. Hypothermia slowed the blood flow and increased the resistance of the microvasculature; however, it did not affect the blood reserve capacity and vascular endothelial function^27^. This treatment achieved good therapeutic effects. Although the types of fillers were different, our study confirmed that a warm compress will contribute to the recovery of intravascular embolism and improve the prognosis compared with a cold compress and no compress. The cold compress seems to have effects similar to those of the warm compress in regard to the thrombus type and hematoma occurrence. The pathological results showed that although a cold compress cannot promote vascular recanalization, it can reduce the white thrombus formed by a large number of PMMA microspheres. These results can indicate whether it is possible to further promote treatment with a warm compress combined with a cold compress; however, further reseach is necessary for confirmation.

In summary, for the first time to our knowledge, our study confirmed that warm compress application after the embolization caused by intravascular injection is conducive to recanalization of vascular embolization and improves tissue necrosis in rabbits. We observed the pathological changes of vascular embolism after the application of the warm compress. However, this study had some limitations. For example, multiple warm compress temperatures and durations were not used; therefore, comparisons of these characteristics could not be performed. Furthermore, the heating rate could have impacted the tissue; however, whether that occurred during our study was not clear. Subsequent research should be performed to further improve the relevant design. This study provides theoretical support for warm compress therapy and further expounds its mechanisms. These results have a guiding role in clinical treatment and create a foundation for additional research in the future.

## Conclusion

Early-stage warm compress after intravascular PMMA injection is conducive to recanalization of vascular embolization and reducing tissue necrosis.

## Data Availability

Data available on request from the authors. The data that support the findings of this study are available from the corresponding author upon reasonable request.
